# Myocardial strain in a health and disease: CMR feature tracking analysis in cardiac hypertrophy

**DOI:** 10.1186/1532-429X-15-S1-E116

**Published:** 2013-01-30

**Authors:** Andrea Barison, Vanessa Cobb, Aye T Hline, Daniel Sado, Steven K White, Andrew Flett, Sanjay M Banypersad, Thomas A Treibel, Anna S Herrey, James Moon

**Affiliations:** 1 Scuola Superiore Sant'Anna, Pisa, Italy; 2 Fondazione G.Monasterio CNR-Regione Toscana, Pisa, Italy; 3 The Heart Hospital Imaging Centre, The Heart Hospital, London, UK; 4 Institute of Cardiovascular Science, University College London, London, UK

## Background

We aimed to study myocardial strain in primary and secondary forms of cardiac hypertrophy, comparing it with normal values from healthy volunteers.

## Methods

Ninety-five healthy volunteers, 45 patients with Anderson-Fabry disease (AFD), 48 with hypertension (HT), 65 with hypertrophic cardiomyopathy (HCM), 62 with severe aortic stenosis (AS), 85 with systemic amyloidosis (AMY) were enrolled. Longitudinal and transversal strain was calculated from 3 long-axis cine views, circumferential and radial strain from 3 short-axis cine images using a dedicated software (Tomtec Feature Tracking). Fourteen healthy volunteers were scanned twice, and inter-study, inter-observer, intra-observer intraclass correlation coefficients ranged between 0.36 and 0.77. Forty AS patients underwent a second scan six months after aortic valve replacement.

## Results

There was a good correlation between ejection fraction (EF) and circumferential (R=-0.781), longitudinal (R=-0.508), transversal (R=0.416) and radial (R=0.367) strain (all p<0.01). Also left ventricular mass index (LVMI) correlated with longitudinal (R=0.464), transversal (R=-0.296), circumferential (R=0.140) and radial (R=-0.140) strain (all p<0.01). AFD and HT did not show significantly reduced global strain values, but AFD presented a reduced basal radial strain, and HT a reduced basal longitudinal strain. HCM showed a reduced global transversal strain: the 36 septal HCM showed also a reduced longitudinal strain in the basal and septal segments, the 23 apical HCM presented a reduced longitudinal strain in the apical and septal segments. AMY presented a reduced global longitudinal and transversal strain, AS a reduced global longitudinal, transversal and radial strain, despite a preserved EF. After aortic valve replacement, global longitudinal, basal circumferential and basal radial strain improved, despite an unchanged EF.

Excluding patients with dysfunction (EF<58%) and/or hypertrophy (LVMI>91 g/m2 for males, >77 g/m2 for females), AFD (n=14) still showed a basal global radial strain , HT (n=31) a reduced basal longitudinal strain, septal HCM (n=6) a reduced basal radial strain, apical HCM (n=6) a reduced apical longitudinal strain, AS (n=13) a reduced global transversal, basal longitudinal and basal radial strain.

## Conclusions

Strain analysis appears to add only modestly to standard parameters (EF, LVMI) in established disease. However,in early disaease or disease post intervention, strain analysis adds incremental information.

## Funding

J.C.M is supported by the Higher Education Funding Council for England.

A.B. is supported by the Scuola Superiore Sant'Anna (Pisa, Italy) and the Fondazione G.Monasterio CNR-Regione Toscana (Pisa, Italy) for a clinical research program on cardiomyopathies.

This work was undertaken at the University College London Hospital and University College London, which receive a proportion of funding from the Department of Health's National Institute for Health Research Biomedical Research Centres funding scheme.

